# Implementación de la figura de agente de apoyo entre iguales en salud mental: una perspectiva internacional en el contexto de su implementación en Cataluña

**DOI:** 10.18294/sc.2023.4252

**Published:** 2023-01-23

**Authors:** Francisco José Eiroa-Orosa, Cecilia Sánchez-Moscona

**Affiliations:** 1 Doctor en Psicología Clínica y Psiquiatría. Investigador Ramón y Cajal, Sección de Personalidad, Evaluación y Tratamiento Psicológico, Departamento de Psicología Clínica y Psicobiología, Universidad de Barcelona. Integrante, Grupo de Investigación en Salud Mental en Primera Persona, Federación Veus, Barcelona, España. fjeiroa@gmail.com Universitat de Barcelona Departamento de Psicología Clínica y Psicobiología Universidad de Barcelona Barcelona Spain fjeiroa@gmail.com; 2 Magíster en Psicología General Sanitaria. Sección de Personalidad, Evaluación y Tratamiento Psicológico; Departamento de Psicología Clínica y Psicobiología, Universidad de Barcelona. Integrante, Grupo de Investigación en Salud Mental en Primera Persona, Federación Veus, Barcelona, España. csanmos@gmail.com Universitat de Barcelona Departamento de Psicología Clínica y Psicobiología Universidad de Barcelona Barcelona Spain csanmos@gmail.com

**Keywords:** Salud Mental, Apoyo entre Iguales, Sistema de Salud, Capacitación de Recursos Humanos en Salud

## Abstract

En el contexto de las discusiones sobre la implementación de la formación y formas de incorporación en el sistema sanitario de Cataluña de agentes de apoyo entre iguales en salud mental o pares, entre 2020 y 2021, se llevó a cabo una revisión de literatura y, de forma complementaria, entrevistas a expertos tanto a nivel internacional como en el Estado español, con el propósito de realizar un análisis de contenido de elementos formativos y de integración dentro de los sistemas sanitarios. Los países germanoparlantes son los que ofrecen programas de formación e incorporación más homogéneos. En el caso de países anglosajones y francófonos, organizaciones sin ánimo de lucro del tercer sector se suelen hacer cargo de los programas formativos y de su incorporación. En el mundo iberoamericano existen diversas experiencias de programas formativos, aunque sin reconocimiento como formaciones sanitarias. Se ofrecen recomendaciones al desarrollo de esta figura en Cataluña, que incluyen el avance hacia una formación profesional con reconocimiento sanitario y opciones de incorporación tanto desde entidades proveedoras sanitarias o sociosanitarias como del tercer sector.

## INTRODUCCIÓN

### El paradigma de recuperación

Para poder comprender de dónde surge el fenómeno de incorporación del apoyo entre iguales a los sistemas de atención en salud mental, debemos situarnos en el paradigma anglosajón de la *recuperación*. La recuperación como movimiento y posteriormente modelo -usamos la palabra paradigma para englobar ambos conceptos- es producto de sinergias entre movimientos de personas afectadas (también autodenominadas como personas con experiencia de sufrimiento psíquico, y, en relación con el sistema de atención, personas usuarias, consumidoras o supervivientes), familiares y profesionales; se define en los inicios de su conceptualización como un “proceso personal, único y multidimensional de cambiar las propias actitudes, valores, sentimientos, metas, habilidades y/o roles que posibilita vivir una vida satisfactoria y esperanzadora, a pesar de las limitaciones causadas por el propio trastorno”^1^. Con raíces en la introducción de medidas de corte humanista después de la Revolución francesa y la guerra civil estadounidense^2^, este movimiento propone desviar el foco de las intervenciones en salud mental más allá de la remisión sintomática. El uso del término recuperación implicaba una polisemia intencionada. Se trata al mismo tiempo de hacer referencia a un proceso subjetivo e idiosincrático, una propuesta de un nuevo objetivo de las intervenciones en salud mental y una estrategia de transformación de servicios a gran escala.

El paradigma de la recuperación nace, entre otras cosas, inspirado por las evidencias de remisión sintomática encontradas en el pionero estudio de Vermont^3^^,^^4^. Los resultados de este estudio longitudinal, confirmado por evidencia producida en las décadas siguientes, ponen de relieve que, así como la remisión sintomática total es posible, incluso en las personas con experiencias de malestar psíquico muy intensas y duraderas, también se puede llevar una vida significativa pese a estar en proceso de recuperación, es decir, quizá experimentando todavía “sintomatología residual”^5^. 

Una de las pruebas del impacto temprano del movimiento de recuperación es la aparición del concepto en el primer informe del “Cirujano General” de EEUU sobre salud mental de 1999^6^. El informe hace hincapié en la participación de las personas afectadas y sus familias en la planificación de servicios y la propuesta de un modelo de atención centrado en “la restauración de la autoestima y la identidad y en el logro de roles significativos en la sociedad”^7^.

Los movimientos de personas afectadas, presentes desde la Inglaterra del siglo XIX, habían reivindicado de distintos modos la humanización de los dispositivos de atención. Exceptuando el cierre de los grandes hospitales y la desinstitucionalización de la atención, las propuestas más revolucionarias, encarnadas sobre todo en el movimiento antipsiquiátrico, se habían dado de un modo marginal. Sin embargo, la aparición y reconocimiento institucional del modelo de recuperación transforma el cuestionamiento del rol de las personas usuarias en la relación asistencial en un debate dominante. En este contexto, las diferentes alianzas de organizaciones profesionales y entidades de representación de personas afectadas y sus familiares se involucran en la trasformación e incluso apropiación de los sistemas de atención. Se propone transitar desde una visión que sitúa a las personas usuarias como sujetos pasivos que deben ser atendidos por el sistema, a una en la que se les considere sujetos de derechos y se les facilite explorar sus fortalezas y apoyar a otras personas en esta búsqueda^8^.

Cabe destacar que, en España y Latinoamérica, hubo algunas diferencias respecto a otros países de Europa occidental en la implementación de las grandes reformas en salud mental. Debido a las dictaduras de ultraderecha presentes en la mayor parte de países de este ámbito durante el siglo XX, la desinstitucionalización del sistema psiquiátrico no comenzó hasta las décadas de 1980 y 1990, con el desarrollo de recursos alternativos a los grandes hospitales psiquiátricos^9).^ Posteriormente, la llegada de influencias del movimiento de recuperación se vio limitada a los servicios de rehabilitación comunitaria, y no fue hasta finales de la década de 2000 cuando se extendió a otros escenarios mediante proyectos específicos.

En el caso español, la crisis de 2008 y los recortes en sanidad que de ella derivaron, llevaron a la retirada de fondos para muchos de estos proyectos^10^. Por otro lado, en lo que respecta a la participación de entidades gestionadas por personas afectadas, el Anteproyecto de Reforma del Código Penal de 2012, impulsado por el gobierno estatal, que proponía la introducción de medidas especiales de seguridad para personas diagnosticadas con trastornos mentales en el Código Penal, causó una reacción inmediata de las principales entidades de salud mental con representación de personas afectadas. Se puede considerar este punto de inflexión como un impulso definitivo del llamado movimiento “En primera persona”. Dentro de este, además de una compleja constelación de asociaciones, existía la Federación “En primera persona” en Andalucía desde 2009 y se formó otra, *Veus*, en Cataluña en 2014. Ambas comunidades también fueron pioneras en la implementación de campañas contra el estigma a nivel autonómico, convirtiéndose en territorios especialmente activos en la lucha por los derechos de las personas diagnosticadas de trastornos psiquiátricos, sin menoscabo de una actividad creciente en el resto del Estado que ha cristalizado en alianzas entre las dos federaciones y el resto de las asociaciones “En primera persona”, así como un comité “En primera persona” dentro de Confederación Salud Mental España, heredera del movimiento de familiares.

En América Latina hay un creciente interés por el modelo de la recuperación^11^ sobre todo como herramienta de trasformación institucional^12^^,^^13^ y reivindicación de ciudadanía^14^ con inspiración en el proceso estadounidense. Los promotores de las ideas de recuperación en América Latina se consideran herederos de movimientos e ideas de arraigo local, como la lucha antimanicomial de Nise da Silveira, la pedagogía de la liberación del brasileño Paulo Freire^15^ o la psicología social de Pichon-Rivière^16^ pionero junto a Harry Stack Sullivan^2^ en la formación de personas usuarias de servicios de salud mental como facilitadoras de la recuperación de otras^17^.

El aterrizaje de estas ideas a los sistemas de gestión sanitaria no ha estado exento de polémicas y ajustes de expectativas, cuando no decepción, de las personas activistas. El colectivo británico “*Recovery in the Bin*” (Recuperación a la Basura) es probablemente el que ha cristalizado de una manera más explícita sus críticas. Acusan al modelo de recuperación de haber sido colonizado y haberse implementado sobre la base de planteamientos neoliberales (por ejemplo, escondiendo recortes de plantilla detrás de “planes autogestionados de recuperación”) de una manera descontextualizada y homogeneizadora que ignora que muchas personas no podrán recuperarse sin antes alcanzar justicia social^18^.

### Apoyo entre iguales

Uno de los elementos distintivos del modelo de recuperación -a veces incluso usado confusamente como metonimia, como si el modelo de recuperación implicase únicamente la incorporación del apoyo entre iguales al sistema^5^- son las intervenciones llevadas a cabo por personas con experiencia de sufrimiento psíquico^19^. Ejemplos de estas intervenciones son la ayuda mutua (definida como la creación y moderación de grupos autogestionados) y el apoyo mutuo o entre iguales (definido como la formación e incorporación al sistema sanitario de personas con experiencia de sufrimiento psíquico). Tanto la ayuda mutua como el apoyo entre iguales consiste fundamentalmente en el apoyo proporcionado a personas con experiencia de sufrimiento psíquico por parte de personas que han tenido experiencias similares. Promueve la recuperación con independencia de diagnósticos o clasificaciones biomédicas y se basa en teorías que consideran que la proximidad social fomenta la motivación^20^, proporciona un referente con quien compararse^21^ y aumenta la comprensión de la propia situación^22^. Este tipo de acompañamiento amplía las redes sociales y ofrece aceptación, apoyo, entendimiento, empatía y sentido de comunidad, lo cual aumenta la esperanza, la autonomía, la autoeficacia y la asunción de responsabilidades^23^. Específicamente, por su rol dentro del sistema, el apoyo entre iguales permite adoptar roles sociales valiosos, ya no restringidos al rol pasivo de la persona usuaria de servicios, sino también como modelos a seguir^24^. Además, puede implementarse a bajo costo y mantiene su utilidad al combinarse con las prácticas profesionales tradicionales^19^. Pese a que la implementación del apoyo mutuo se ha enfocado hasta ahora en poblaciones adultas, también empiezan a haber programas enfocados a la población infanto-juvenil^25^.

Entre los elementos claves de la formación de esta figura profesional denominada internacionalmente “par” (*peer*), un estudio Delphi ha identificado los siguientes: experiencia vivida como recurso, práctica ética, bienestar propio, enfoque en la recuperación y capacidad de comunicación^26^. Una vez incorporadas, las tareas que realizan estas personas pueden categorizarse como apoyo directo o indirecto^27^. Las actividades consideradas tareas directas son la defensa de derechos (facilitar a la persona acompañada información y soporte), la conexión a recursos (conectarla con los servicios deseados), compartir experiencias comunes, construir comunidad (conectarla con programas que la enlacen a otras personas), construir relaciones (basadas en la confianza), la facilitación de actividades grupales, el desarrollo de habilidades y objetivos, la socialización y el desarrollo de la autoestima. Las tareas indirectas incluyen tareas administrativas, comunicación, supervisión, formación, recepción de apoyo, y la obtención y verificación de información. Además, la labor de las y los agentes de apoyo entre iguales también incluye acciones dirigidas a construir relaciones con los profesionales y a legitimar el rol de la figura^28^. De esta forma, las herramientas que requieren los pares para desempeñar sus funciones van más allá de la experiencia de sufrimiento psíquico e incluyen la experiencia vital de recuperación y resiliencia, el acercamiento respetuoso, la presencia genuina, el modelaje, la colaboración y el compromiso^27^.

### Dificultades y polémicas sobre su implementación

Desde los sistemas de atención se han identificado cuestiones problemáticas asociadas al apoyo entre iguales como, por ejemplo, la dificultad de integración en los equipos de trabajo, la presencia de actitudes negativas hacia el modelo de recuperación por parte de otros profesionales, el conflicto y confusión de roles, la falta de políticas y prácticas con respecto a la confidencialidad, estructuras y culturas organizativas sin suficiente definición y la falta de apoyo y de trabajo en red^29^^,^^30^. Como estrategias para mejorar estos aspectos, se recomienda establecer estructuras, políticas y prácticas que guíen la incorporación de la figura y que ayuden a definir de manera más clara su rol^29^^,^^31^^,^^32^, así como asegurar la supervisión y el acompañamiento de otros profesionales, tanto de apoyo mutuo como del ámbito clínico clásico^33^^,^^34^.

Desde los movimientos de personas afectadas, las dudas en torno a la implementación del apoyo entre iguales como figura profesionalizada tienen que ver con la problematización de la adopción del rol de “expertas” o personas que “poseen un saber experto” contraponiéndolo a la propia experiencia vivida o a los “saberes profanos”^35^. En otras palabras, si la inclusión de estas personas en el sistema se planteaba como una punta de lanza para fomentar reformas a través de un trabajo que reconozca el valor de la experiencia y la horizontalidad en el trato, si muchas de estas personas acaban adoptando una identidad profesional similar en su verticalidad a la de las y los profesionales tradicionales, el cambio se habrá obrado en la persona, que se habrá adaptado al sistema tal y como está^32^. En este caso, la inclusión de pares podría incluso ayudar a apuntalar el sistema de atención, ya que se podrá justificar que se han hecho “cambios” cuando en realidad todo sigue igual. Como contrapartida, también se argumenta que conservar una identidad muy vinculada al pasado como personas usuarias de servicios puede alejar a las y los agentes de una identidad profesional necesaria para la integración dentro de los equipos profesionales^36^. Como síntesis, en una reflexión sobre una experiencia pionera en Argentina^37^ se plantea el posible aporte de la incorporación de pares a los equipos de salud mental como una ayuda a los profesionales para que puedan reflexionar sobre la dimensión relacional de su tarea, y sobre sus formas de tratar y pensar acerca de las personas usuarias. 

A nivel científico, la supuesta escasez de evidencia empírica que respalde la efectividad y eficacia del apoyo entre iguales es uno de los argumentos principales de las personas que no apoyan su implementación. Sin embargo, existen un total de nueve trabajos de revisión y síntesis cuantitativa^38^^,^^39^^,^^40^^,^^41^^,^^42^^,^^43^^,^^44^^,^^45^^,^^46^, si bien es cierto que seis de ellos han sido publicados desde 2019. Las aproximaciones de estos trabajos (por ejemplo, comparaciones con otras profesionales, como gestores de casos, o entre añadir o no añadir la figura) y las variables que abordan, clasificadas frecuentemente en torno a la sintomatología, el uso de servicios o la subjetividad (recuperación, esperanza y empoderamiento) son muy distintas. En todos los casos, sin excepción, se hace alusión a la baja calidad de los estudios y alto riesgo de sesgos. Las recomendaciones varían entre las críticas feroces a la implementación del apoyo entre iguales profesionalizado sin que exista una evidencia clara^40^^,^^45^, la neutralidad^38^^,^^42^^)^ y un cauto apoyo^39^^,^^41^^,^^43^^,^^44^, con la notable excepción del metaanálisis más reciente, de mayor calidad y que incluye más estudios cuyas conclusiones apoyan, sin más matices que la apuesta por estudios de más calidad (coletilla omnipresente en prácticamente cualquier metaanálisis de intervenciones psicosociales), la implementación de la figura^46^. La conclusión más extendida en todos estos estudios es que el apoyo entre iguales solo tendría efecto sobre variables de recuperación, esperanza y empoderamiento, pero no de sintomatología, uso de servicios o salud física^39^^,^^40^^,^^41^^,^^42^^,^^44^^,^^45^^)^ con la excepción de uso de servicios de emergencia^38^^)^ y sintomatología depresiva^43^. Este argumento habría sido refutado por el trabajo más reciente, publicado en septiembre de 2022, que confirma la eficacia en términos tanto de sintomatología como de recuperación, esperanza y empoderamiento, aunque no funcionalidad^46^.

La polémica en torno a la evidencia empírica del apoyo entre iguales genera discusiones incluso en regiones como América del Norte, donde la implementación está avanzada. En un artículo de opinión de 2018, el muy mediático y recientemente fallecido experto en legislación sobre salud mental DJ Jaffe^47^ argumenta que muchos de los estudios sobre la eficacia del apoyo entre iguales profesionalizado han sido realizados por las propias entidades que ofrecen programas, que muchos no cuentan con rigor experimental y no indican el diagnóstico o la severidad de los trastornos de las personas a las que se atiende. De este modo, Jaffe argumenta que tampoco se puede concluir qué valor tiene este tipo de apoyo en personas con “trastornos mentales severos” haciendo una clara distinción con personas con otros niveles de afectación. Asimismo, haciendo alusión a la comparación entre agentes de apoyo entre iguales con profesionales en roles similares, realizada por la colaboración Cochrane^38^, plantea la cuestión de si los resultados favorables de algunos estudios pueden justificarse por la mera presencia de una persona independientemente de su formación u orientación. Además, se critica que en la mayoría de los estudios que avalan el apoyo mutuo profesionalizado solo informan mejoras en “variables blandas” como esperanza, autoestima o empoderamiento, sin hablar de las “variables duras y significativas” que según el autor serían las tasas de falta de vivienda, arresto, hospitalización innecesaria, encarcelamiento, suicidio y victimización.

En respuesta a este artículo, un grupo de defensores del apoyo entre iguales profesionalizado liderados por el catedrático de la Universidad de Yale Larry Davidson^48^ consideran que hablar de falta de evidencia sería impreciso, ya que en 2018 más de 30 estudios habían encontrado resultados positivos en diversas áreas. A modo aclaratorio, establecen que hay una gran diferencia entre tratar un trastorno y ayudar a alguien que está en un proceso de recuperación y que las personas que viven con un sufrimiento psíquico significativo requieren de más asistencia y apoyo que la que se puede brindar a nivel estrictamente biomédico. Consideran, además, que el hecho de que el despliegue de apoyo entre iguales aumente el uso de servicios de crisis y disminuya las hospitalizaciones, aumentando con ello algunos costos^49^, debe interpretarse de forma favorable, ya que de lo contrario muchas de estas personas habrían quedado descolgadas de la atención ambulatoria y con ello, aunque disminuyan los costos sanitarios, aumentarían significativamente los costos sociales. Capacitar y contratar a personas en recuperación para apoyar a otras supone un beneficio para todas las partes en sistemas fragmentados con escasos recursos: las personas usuarias reciben apoyo de profesionales que fomentan la esperanza y les ayudan a navegar por el sistema de salud, estos profesionales tienen un empleo remunerado en un rol que apoya su propia recuperación y los sistemas obtienen personal cualificado que impulsa la obtención de resultados asociados a la recuperación de una vida significativa y no a la reducción de síntomas. El rol de agente de apoyo entre iguales no incluye tratar la propia sintomatología o suplir las funciones de otros profesionales, sino que busca complementar la atención clínica, sin minimizar la importancia de que se aborden aspectos como el encarcelamiento, la hospitalización o la falta de vivienda. La evidencia en este sentido enfatiza que las y los agentes ayudan a involucrar a las personas en relaciones afectivas; a mejorar las relaciones entre personas usuarias y proveedores de servicios ambulatorios, disminuyendo su asistencia a servicios de emergencia y el costo de la atención; a disminuir el uso de sustancias y a aumentar la esperanza, el empoderamiento, la autoeficacia, el autocuidado y la calidad de vida.

Aparte de estas discusiones que tienen lugar en América del Norte, desde nuestra experiencia incipiente en Cataluña nos parece importante destacar que la exigencia de la demostración de la eficacia de una profesión, y no de sus intervenciones, es inédita en el campo de la salud. Hoy en día, nadie pondría en duda la utilidad de una profesión sanitaria, sino que se evalúan sus intervenciones concretas y no su existencia en general. Por ello creemos que, con el apoyo entre iguales, el nivel de exigencia debería ser similar: evaluar sus intervenciones, no su existencia. De lo contrario, lo que se está haciendo probablemente es mezclar cosas muy distintas. Es probable que algunas aproximaciones al trabajo entre pares funcionen mejor que otras, pero dudar de las ventajas de empoderar a las personas afectadas, independientemente del efecto de eso en variables consideradas “duras” tiene importantes implicaciones ideológicas en la justificación de un sistema coercitivo y vertical.

### Objetivo

El objetivo de este artículo es identificar y describir las experiencias existentes de implementación de la figura del agente de apoyo entre iguales en salud mental a nivel internacional, pero con un foco especial en el Estado español; ya que, aparte de su valor académico, desarrollamos este trabajo en el contexto de la preparación de posibles programas de implementación en Cataluña. 

## MÉTODOS

Como punto de partida, entre 2020 y 2021, se llevó a cabo una revisión de literatura sobre la implementación de la figura del agente de apoyo entre iguales, en diversos países, mediante búsquedas en Google con el objetivo de encontrar literatura gris, y en las siguientes bases de datos con el objetivo de encontrar artículos revisados por pares: Google Scholar, Scopus, APA PsycInfo y Medline. Los términos de búsqueda utilizados incluyeron: apoyo mutuo, apoyo entre iguales, implementación, incorporación, integración, contratación y formación. Las búsquedas se realizaron en alemán, castellano, catalán, francés, italiano e inglés. Para ser incluidos en la revisión, los documentos debían abordar la formación y/o incorporación de la figura profesionalizada del agente de apoyo entre iguales en uno o varios sistemas de salud. Por motivos de exhaustividad y espacio no incluimos información sobre grupos de ayuda mutua u otras iniciativas autogestionadas independientes a los sistemas de salud.

En paralelo, con el objetivo de complementar la revisión en los casos en los que faltaba información, se enviaron correos electrónicos a actores claves de la implementación de la figura de agente de apoyo entre iguales a nivel internacional. A partir de las respuestas obtenidas y mediante el método de *bola de nieve*, procedimos a entrevistar a diversos expertos (ver agradecimientos), por teléfono o por correo electrónico, sobre el tipo de incorporación y otros factores relacionados con la implementación de la figura de agente de apoyo entre iguales en los sistemas sanitarios de sus países. A partir de toda la información extraída de estas fuentes, se procedió a realizar un análisis de contenido. El criterio para extraer contenido fue aportar información sobre los sistemas de formación e incorporación al sistema sanitario de la figura de par o agente de apoyo entre iguales.

Todos los documentos analizados son referenciados en la sección correspondiente. El listado de personas entrevistadas puede encontrarse en la sección de agradecimientos.

## RESULTADOS

El resumen de los resultados puede encontrase en la Tabla 1. Diversos países cuentan con programas de formación de apoyo entre iguales con diferentes niveles de reconocimiento y acreditación sanitaria. No obstante, el mayor reto sigue siendo la incorporación de esta figura dentro de los sistemas sanitarios.

**Tabla 1:** Síntesis de los programas de formación de apoyo entre iguales, según país o región. Años 2020-2021

País o región	Formación	Instituciones acreditadoras formación	Denominación	Incorporación
Canadá	Curso certificado	ONG	Peer support worker, recovery specialist	ONG, Proveedores sanitarios
EEUU	Curso certificado + examen estatal	ONG, Universidades	Peer support worker, recovery specialist	ONG, Proveedores sanitarios
Reino Unido e Irlanda	Curso certificado por el Sistema Nacional de Salud	ONG, Sistema Nacional de Salud	Peer support worker, recovery coach	ONG, Servicio Nacional de Salud
Australia	Curso certificado	ONG, Universidades	Peer support worker, recovery specialist	ONG, Proveedores sanitarios
Nueva Zelanda	Curso certificado	ONG, Universidades	Peer support worker	ONG, Proveedores sanitarios
Italia	Heterogéneo. Existen cursos oficiales.	ONG	Esperti in supporto tra pari	ONG, Proveedores sanitarios
Países germanoparlantes	Formación profesional oficial	ONG (ExIn), consorcio europeo (UPSIDES)	Genesungsbegleiter (acompañante de recuperación), peer-begleiter (acompañante par)	Proveedores sanitarios
Escandinavia	Curso certificado	ONG	Erfaringskonsulent (consultor con experiencia, Dinamarca, Noruega), Kamratstödjare (compañero de apoyo, Suecia)	ONG, Proveedores sanitarios
Francia, Bélgica y Suiza francófona	Muy heterogéneo, algunos cursos certificados	ONG – Universidades	Médiateur de santé pair (Francia), pair aidant (Francia, Bélgica), expert du vécu (Bélgica), pair praticien (Suiza)	ONG, Proveedores sanitarios
Países Bajos	Curso certificado	ONG	Ervaringsdeskundigen (expertos por experiencia)	ONG, Proveedores sanitarios
Argentina	Muy heterogéneo, en general no oficial	ONG	Agente de apoyo mutuo, par	ONG
Brasil	Muy heterogéneo, en general no oficial	ONG	Trabalhador/a de apoio de pares	ONG, Proveedores sanitarios
Chile	Muy heterogéneo, en general no oficial	ONG	Especialista en apoyo a pares	ONG
España	Muy heterogéneo, en general no oficial	ONG	Agente, técnico/a de (acompañamiento y) apoyo entre iguales, apoyo mutuo	ONG, Servicio Nacional de Salud (de manera muy limitada)

Fuente: Elaboración propia.

Hay seis proyectos internacionales que merecen mención aparte. En primer lugar, el proyecto *Empowerment of Mental Illness Service Users: Lifelong Learning, Integration and Action - EMILIA*^50^, parte del sexto programa marco de investigación de la Comisión Europea, fue implementado entre los años 2005 y 2010. En él participaron equipos de Bosnia-Herzegovina, Eslovenia, España, Grecia, Dinamarca, Finlandia, Francia, Lituania, Noruega, Suecia, Polonia y Reino Unido. Una de las actividades del proyecto incluía la formación de personas usuarias “expertas”. Aunque este concepto no coincide totalmente con la definición que usamos en este trabajo de apoyo entre iguales, sí que es uno de sus antecedentes más inmediatos. La implementación del proyecto supuso la apertura de dos líneas de implicación laboral: la de la persona “experta” como formadora de personas usuarias y como mediadora entre las instituciones sanitarias y las personas usuarias^51^^,^^52^.

En segundo lugar, los proyectos *Experienced Involvement - EX-IN*^53^ y *Peer to Peer: A route to recovery of people with mental illness through peer support training and employment, acortado peer2peer*^54^, del programa Leonardo da Vinci (integrado en la actualidad dentro del macroprograma Erasmus+). El primero, más centrado en el ámbito germanoparlante, será comentado en el correspondiente encabezado territorial. En cuanto al proyecto peer2peer, integrado por equipos de Austria, España, Escocia (Reino Unido) y Rumania, supuso la primera implementación a nivel europeo del marco anglosajón de apoyo entre iguales. Como proyecto de formación continua, sus actividades estuvieron muy centradas en la creación de un marco común de formación^55^ adaptado del programa Professional Development Award (PAD) in Mental Health Peer Support desarrollado por la Scottish Recovery Network, una de las entidades que lideró el consorcio.

Más recientemente, la iniciativa *UPSIDES* (*Using Peer Support in Developing Empowering Mental Health Services*), a través de financiación del Octavo Programa Marco de investigación de la Comisión Europea, Horizonte 2020, ha establecido una comunidad internacional de investigación y práctica de apoyo entre iguales^56^. El proyecto es un estudio llevado a cabo en seis países durante cinco años, que replica y amplía las intervenciones de apoyo entre iguales, generando evidencia de prácticas sostenibles en países de ingresos altos, medios y bajos (dos centros tanto en Alemania como en el Reino Unido; y un centro en cada uno de los otros cuatro países participantes: India, Israel, Tanzania y Uganda).

Por último, los proyectos financiados dentro del programa Erasmus+, actualmente en funcionamiento *Peer Support+*, que incluye socios de Estonia, Islandia y Países Bajos, y *TuTo3-PAT - Peer and Team Support,* que incluye socios de Alemania, Bélgica, España, Francia, Noruega, Quebec (Canadá) y Rumania. El primero está centrado en el desarrollo de materiales formativos en metodologías de “apoyo experiencial” y ha sido desarrollado entre 2020 y 2022. El segundo, inaugurado en 2022, pretende ir más allá de los resultados del proyecto *peer2peer*, define un marco competencial y formativo (tanto de las personas afectadas como de los equipos en los que se integran), que facilite el reconocimiento profesional a nivel europeo e internacional.

A continuación, expondremos el panorama actual de diversos países en que la figura de apoyo entre iguales está en proceso de implementación. Intentamos combinar un orden cronológico con uno territorial, exponiendo primero, en la medida de lo posible, regiones donde la aparición de la figura fue más temprana. En territorios donde la implementación es reciente, como Iberoamérica, seguimos un orden alfabético.

### Países anglosajones

#### Canadá

En Canadá, país pionero en la implementación de apoyo entre iguales en el sistema de apoyo a las personas con discapacidad, existe un proyecto llamado *Peer Support Canada*^57^, vinculado con la asociación de Salud Mental Canadiense, encargada de la certificación profesional de los pares, y la Comisión de Salud Mental de Canadá^58^, que ha publicado guías de práctica para el apoyo entre iguales^59^ y monitoriza su implementación e impacto en el sistema de salud^60^.

A nivel de formación, Canadá cuenta con un programa oficial reconocido por los principales proveedores de salud mental a nivel federal. Este país cuenta con unos estándares de prácticas nacionales aprobados y establecidos que incluyen conocimientos, competencias, experiencia y requisitos del código de conducta para proporcionar de forma efectiva servicios de asistencia entre iguales^57^. En cuanto a la incorporación, hay una serie de requisitos que incluyen haber cursado dicha formación, la consulta de antecedentes penales y haber realizado prácticas adicionales.

Por otro lado, Canadá cuenta con iniciativas locales como la *Ontario Peer Development Initiative* (OPDI), previamente conocida como *Consumer Survivor Development Initiative* (CSDI), fundada el 1991^61^. Este proyecto ofrece una formación sólida para las y los agentes de apoyo entre iguales, ya que sus experiencias han mostrado la importancia de establecer un estándar mínimo de capacitación de este tipo, sobre todo en áreas rurales^62^.

#### Estados Unidos de América

EEUU es otro veterano en la implementación de programas de apoyo mutuo en su complejo sistema sanitario. La mayoría de los estados han establecido ya programas para formar y certificar a las y los agentes de apoyo entre iguales o están en proceso de implementar un programa según muestra un sistema de monitorización de la Universidad de Texas^63^^,^^64^. Por un lado, los programas de formación difieren entre estados; pero, en su mayoría, están a cargo de entidades gestionadas por personas con experiencia de sufrimiento psíquico y disponen de exámenes de certificación propios. Así, los programas formativos son obligatorios para poder obtener la certificación correspondiente, y también lo son para poder pedir reembolsos a través de *Medicaid,* el seguro del gobierno destinado a personas cuyos ingresos y recursos son insuficientes para pagar la atención médica^63^. Por otro lado, la incorporación se lleva a cabo por agencias específicas que realizan contrataciones, utilizando categorías laborales paraprofesionales genéricas que no requieren licencias otorgadas por los estados, como es el caso del resto de profesiones sanitarias.

#### Reino Unido e Irlanda

El apoyo entre iguales es una profesión reconocida tanto en Irlanda como en el Reino Unido, aunque de manera distinta en cada una de sus naciones constituyentes. Hay organizaciones sin ánimo de lucro (*charitable bodies*) muy establecidas, que fomentan la formación y la incorporación como la Mental Health Foundation o la Scottish Recovery Network. En el caso escocés, por ejemplo, mientras que durante un tiempo la formación de apoyo entre iguales en Escocia estuvo a cargo de un proveedor estadounidense Recovery Innovations, con el objetivo de promover la perdurabilidad y su adaptación al contexto nacional, la Scottish Recovery Network trabajó con la autoridad nacional de cualificaciones sanitarias (Scottish Qualifications Authority*)* en el desarrollo de una acreditación nacional^23^. Así, en la actualidad, los programas formativos son llevados a cabo por organizaciones independientes como la Scottish Recovery Network, la cual cuenta con validez dentro del marco escocés de acreditación y cualificaciones, provisto por la Scottish Qualifications Authority^23^^,^^65^. Dicha formación se compone de dos unidades: una teórica (*recovery context*), basada en los conceptos de recuperación, empoderamiento, mutualidad y el rol de las y los agentes de apoyo entre iguales; y una práctica (*developing practice*), que pretende acercar a los participantes de una forma más aplicada a todos los conocimientos, habilidades y valores requeridos para su práctica^66^. La incorporación se lleva a cabo, mayoritariamente, en el tercer sector, a través de organizaciones no gubernamentales (ONG) y proveedores de atención social. Además, la Scottish Recovery Network estuvo implicada en el ya mencionado proyecto *peer2peer*^54^, que dio paso a programas formativos en otros lugares.

Cabe destacar que, en el Reino Unido, se han desarrollado programas de apoyo mutuo en población infanto-juvenil. Como ejemplo hallamos la institución Nottingham Healthcare NHS Foundation Trust, la cual ha comenzado a desarrollar un proyecto de apoyo mutuo entre personas usuarias de sus centros de atención a la salud mental infanto-juvenil (CAMHS, por sus siglas en inglés), obteniendo resultados satisfactorios^25^.

En Irlanda, aunque la incorporación de agentes de apoyo entre iguales ha sido más reciente, actualmente está totalmente normalizada, contando con certificaciones de las universidades de la Ciudad de Dublín y la Tecnológica Atlántica y contrataciones en el sistema de salud^67^. La oficina de salud mental del Servicio Nacional de Salud (Health Service Executive) ha publicado recientemente un extenso informe sobre su impacto en los servicios de atención a la salud mental^68^.

#### Australia y Nueva Zelanda

En ambos países la implementación de la profesión está muy avanzada. Los programas formativos están generalmente a cargo de ONG. Respecto a las contrataciones, en ambos países existen categorías profesionales específicas y hay posibilidades laborales, tanto a través del tercer sector como de los sistemas de salud mental. En Nueva Zelanda, la formación de apoyo entre iguales es actualmente un certificado de nivel 4 en el marco nacional de cualificaciones. En la Universidad Tecnológica de Auckland, en 2023, comenzará un nuevo programa académico que conducirá a un grado en liderazgo con experiencia propia. Adicionalmente, una institución dedicada al desarrollo de recursos humanos de Nueva Zelanda ha publicado un documento con una estrategia clara de implementación del apoyo entre iguales entre 2020 y 2025^69^.

#### Sudáfrica

En Sudáfrica la implementación de la figura es todavía incipiente. Existen recientes estudios sobre las fortalezas y limitaciones del proceso de introducción de pares en el sistema de atención^70^.

#### Israel

Aunque no sea un país formalmente angloparlante, Israel es un país donde el apoyo entre iguales tiene un gran arraigo, incluyendo un coordinador de implementación en el Ministerio de Sanidad, siguiendo un modelo de formación e incorporación similar al de los países anglosajones^71^.

#### Italia

Italia tiene su propio modelo de salud mental comunitaria con raíces en los movimientos antipsiquiátricos de mediados del siglo XX. Aunque guarda similitudes con el modelo de la recuperación, Italia fue pionera en los procesos de desinstitucionalización y su modelo se puede considerar más bien como un antecedente. En los últimos años, en regiones como Lombardía, se han desarrollado experiencias de formación de personas usuarias y apoyo entre iguales a partir de formas de gestión colaborativa entre cooperativas y el sistema sanitario^72^. Actualmente, unos cien agentes prestan su servicio en una decena centros de salud mental lombardos, pagados directamente con fondos de la región o a través de convenios con cooperativas proveedoras de servicios^73^. Otros lugares, clásicos en el modelo de salud mental comunitaria italiano como Trieste, también han incorporado agentes de apoyo^74^. En 2021, se celebró el primer Congreso Nacional de Personas Usuarias y Familiares Expertas en Apoyo entre Pares, que ha dado pie a la publicación de un documento nacional de apoyo entre iguales en salud mental patrocinado por el Ministerio de Salud que define competencias y funciones de la figura^75^. 

#### Países germanoparlantes: Alemania, Austria y Suiza

Los tres países germanoparlantes cuentan con entidades derivadas del ya mencionado proyecto EX-IN^76^^,^^77^^,^^78^, implementado entre 2005 y 2007. Estos países han desarrollado un programa integrado en sus sistemas de formación profesional. El currículum del programa fue desarrollado de forma cooperativa entre personas usuarias y profesionales de servicios de salud mental, investigadores y formadores y está dirigido por formadores certificados. Adicionalmente, la incorporación ha sido integrada de forma oficial a los sistemas de provisión de servicios sanitarios de los tres países germanoparlantes. A esta sólida línea formativa y de incorporación se ha añadido recientemente el brazo alemán del consorcio UPSIDES con presencia en tres ciudades alemanas^79^.

#### Países Nórdicos: Escandinavia y Finlandia

Aunque la implementación en estos países se ha materializado sobre todo en la década de 2010, los cinco países nórdicos (Dinamarca, Finlandia, Islandia, Noruega y Suecia) cuentan con una extensa red de agentes de apoyo entre iguales y programas formativos reconocidos oficialmente. En general, en todos los países de la región la formación y contratación se hace a través de organizaciones independientes, pero las y los agentes son integrados en las redes de servicios sociales y atención a la salud mental.

En Dinamarca existen iniciativas desde la década de 1990, aunque hasta 2010 no se hicieron pilotajes de experiencias de implementación global en las zonas más pobladas^80^. En 2014, se publicó un extenso mapeo que recogía los proveedores de servicios de apoyo entre iguales en todas las regiones del país^81^ y, en 2018, una propuesta de marco común de formación^82^. Además, se ha implementado un ambicioso programa a nivel estatal de apoyo entre gente joven experimentando ansiedad y depresión^83^.

Finlandia introdujo en 2009 la obligatoriedad de la participación de expertos con experiencia y agentes de apoyo entre iguales en la planificación y desarrollo de servicios, como uno de los objetivos principales de su Plan Nacional de Salud Mental y Abuso de Sustancias^84^. Además, el modelo de Diálogo Abierto, originado en la región finlandesa de Laponia, pero de amplísima difusión internacional en los últimos años, ha incluido agentes de apoyo entre iguales en los equipos^85^. Islandia, el más pequeño -tanto por tamaño como por población (apenas 370.000 habitantes)- tiene entidades totalmente dedicadas al apoyo entre iguales desde principios de la década de 2000 con un programa formativo propio, que están reforzando con socios neerlandeses y estonios en el contexto del proyecto Erasmus+ *Peer Support+*^86^.

Desde 2012, Noruega también cuenta con directrices nacionales que apoyan la introducción de la figura de agente de apoyo entre iguales. Con academias oficiales de formación en varias regiones, un mapeo de necesidades^87^, un manual para la correcta contratación^88^, una red de defensa de intereses y organización de actividades^89^ y una sólida implementación a través de financiación municipal, es un país con una sólida apuesta por el apoyo entre iguales profesionalizado con niveles de impacto en el sistema de atención comparables a países veteranos como Canadá^90^.

Por último, Suecia también cuenta con un modelo oficial de formación y contratación^91^ promovido por la entidad de personas usuarias Colaboración Nacional para la Salud mental (NSHP, por sus siglas en Sueco), que impulsa un proyecto para la integración de la figura en el sistema de salud desde 2016^92^. 

#### Países europeos francófonos: Francia, Bélgica y Suiza

El panorama francófono es muy heterogéneo^93^^,^^94^. Francia cuenta con diversos programas formativos en distintas regiones^95^. Existen varios programas formativos acreditados con reconocimiento en todo el estado, como el programa *Médiateur de Santé/Pair* llevado a cabo por el Centro Colaborador de la Organización Mundial de la Salud para la Investigación y la Formación en Salud Mental (CCOMS Lille, por sus siglas en francés), o los diplomas universitarios de la Université Paris 8 (Vincennes - Saint-Denis) y Université Paris 13 (Bobigny), Université Grenoble Alpes, Aix-Marseille Université o Université Claude Bernard Lyon 1^96^. Otras opciones incluyen organizaciones sin ánimo de lucro como la red de cuidados *Solidarités Usagers Psy*.

El programa formativo del centro colaborador de la Organización Mundial de la Salud es uno de los más completos a nivel internacional y contó con una fase piloto en la que se tuvieron en cuenta todo tipo de aspectos, desde los contractuales a las experiencias subjetivas de las y los pares y sus equipos^95^. Se lleva a cabo en dos fases, ambas de un año de duración. La primera consiste en una formación universitaria certificada y la segunda en la integración en los equipos a través de prácticas. De la teoría se hace cargo la Université de Lille y las sesiones formativas se llevan a cabo en tres regiones. La formación práctica implica a los 16 servicios de psiquiatría de adultos que acogen mediadores/compañeros de salud en formación. La inscripción a la formación está sujeta a la incorporación en uno de estos 16 departamentos, de manera que los participantes de este programa tienen un contrato profesional de dos años, en forma de contratos de duración determinada renovables. Como parte de esa capacitación, los pares realizan prácticas a tiempo completo o parcial y trabajan junto a un profesional del equipo.

En cuanto a Suiza, aparte del modelo EX-IN de la parte germanoparlante, la parte francófona, con una incipiente implementación de la figura^32^, tiene programas formativos oficiales como el liderado por la entidad sin ánimo de lucro Re-pairs^97^ de dos años de duración. Por su parte, en las regiones mayoritariamente francoparlantes belgas de Valonia y Bruselas se han desarrollado múltiples iniciativas de formación e incorporación de pares que implican una tupida red de apoyo^98^. Existe una cartografía de todas las entidades dedicadas al apoyo entre iguales que, a la fecha de cierre de este artículo, cuenta con 35 organizaciones, de las cuales 12 están especializadas en salud mental^99^. Tres entidades locales (la ONG En Route, el hospital neuropsiquiátrico San Martín y la Escuela Superior de Namur) lideran el ya mencionado proyecto TuTo3-PAT del programa Erasmus+, actualmente en ejecución.

#### Países Bajos

En los Países Bajos, el Instituto Nacional Trimbos y la organización nacional de salud mental GGZ Nederland diseñaron un perfil de competencia profesional para las y los agentes de apoyo entre iguales en 2013. En 2015, se publicó un currículum oficial de formación^100^. Actualmente, las autoridades neerlandesas de atención sanitaria están acreditando una profesión reconocida y un puesto formal para las y los agentes que trabajan en entornos de atención sanitaria^101^.

#### Rumanía

Como ya comentamos anteriormente, personas de Rumania participaron y participan en dos de los grandes proyectos europeos sobre apoyo entre iguales, *peer2peer* (2013-2015) y TuTo3-PAT (2022-2025). A pesar del éxito del primero, que implicó actividades formativas en Rumania^102^ y la traducción del manual al rumano^103^, no se tiene constancia de la continuidad de planes de implementación.

#### Latinoamérica: Argentina, Brasil, Chile

Argentina cuenta con diversas experiencias y reflexiones en torno a la implementación del apoyo entre iguales profesionalizado desde principios de la década de 2010^33^. Por ejemplos, el proyecto de la ONG Suma^33^^,^^37^^)^ y la Fundación Bipolares de Argentina, que lleva a cabo un programa formativo basado en los materiales elaborados por el proyecto *peer2peer*^104^. Sin embargo, no parece haber un marco claro de incorporación al sistema de salud.

Otro país con experiencias de formación de pares es Brasil, que cuenta con distintas iniciativas de formación^105^. Aunque todavía no existe un sistema oficial de legitimación de la figura, algunas organizaciones independientes lo hacen a su propia discreción y pueden o no estar conectadas con agencias gubernamentales u otras organizaciones que pueden reconocer y contratar a las y los agentes de apoyo entre iguales. En algunos estados, las y los agentes son contratados por organizaciones gubernamentales. Sin embargo, y aunque el proceso ha sido iniciado, aún no se dispone de una categoría específica dentro del sistema brasileño de clasificación de ocupaciones (CBO, por sus siglas en portugués).

Chile, con experiencias en procesos de investigación gestionados por personas afectadas^106^, es otro país iberoamericano con experiencias en apoyo entre iguales, aunque aparentemente de manera puntual, sin una coordinación a nivel nacional. Existen experiencias vinculadas a entidades sin ánimo de lucro de asociaciones de personas usuarias y sus familiares^107^ en colaboración con agrupaciones profesionales^108^.

#### Estado Español

Dentro de España, foco principal de este trabajo, hallamos diferentes experiencias de formación e incorporación de agentes de apoyo entre iguales. Recientemente, se ha llevado a cabo una revisión bibliográfica sobre las experiencias de formación basadas en el apoyo entre iguales en salud mental realizadas en España^109^. Se identificaron cinco experiencias que serán explicadas en sus correspondientes encabezados territoriales. 

A nivel estatal, la Confederación Salud Mental de España, ha llevado a cabo dos ediciones del curso “Informando en primera persona”, el cual pretende dotar de herramientas e información a pares voluntarios para que puedan acoger y acompañar a otras personas afectadas en las entidades a las que pertenecen. Cuenta además con una evaluación que se realiza al finalizar la formación y una valoración de la utilidad de la formación a los seis meses de concluirla^109^. 

A continuación, haremos un recorrido por aquellas comunidades autónomas en que la figura de agente de apoyo entre iguales está en proceso de implementación.

#### Andalucía

La primera formación de pares implementada exclusivamente en España se llevó a cabo en Andalucía entre los años 2010 y 2011. Fue a través del proyecto “Ayuda Mutua en los Servicios de Salud Mental de Andalucía”, el cual se enmarcó en el II Plan Integral de Salud Mental de Andalucía y fue coordinado por la Escuela Andaluza de Salud Pública y la Federación Salud Mental Andalucía^109^.

También en Andalucía se encuentra la Federación “En primera persona” que da apoyo a la Red de Apoyo Mutuo de Andalucía (RAMA). La Federación “En primera persona” estuvo implicada en el proyecto *peer2peer*^54^, liderado por la fundación castellano-leonesa Intras y la Scottish Recovery Network. El programa formativo se plantea de forma práctica y participativa y cuenta con una evaluación intermedia y otra al final^109^. Posteriormente, se han realizado contrataciones de corta duración a través de asociaciones federadas en el sistema Andaluz de Salud^110^.

#### Castilla-La Mancha

Castilla-La Mancha ha sido la comunidad autónoma pionera en insertar laboralmente la figura profesional de expertos por experiencia. Desde el 2018, esta comunidad autónoma cuenta con el Proyecto Experto por Experiencia de la Fundación Sociosanitaria de Castilla-La Mancha, de carácter autonómico^111^. El objetivo del proyecto es integrar a personas con experiencia de sufrimiento psíquico como profesionales del equipo de los Centros de Rehabilitación Psicosocial y Laboral (CRPSL). Se ha implementado un programa formativo que ha dado lugar a contrataciones a través del Plan Extraordinario por el Empleo en Castilla-La Mancha. Cabe destacar que el programa formativo cuenta con una fase final de supervisión de las y los agentes de apoyo entre iguales contratados por los CRPSL^109^.

#### Castilla y León

Esta comunidad autónoma se incorporó al proyecto europeo *peer2peer*^54^ a través de la fundación Intras. Esta entidad ha seguido haciendo actividades formativas, tanto en Castilla y León como en otras comunidades, como Andalucía o Baleares. Además, desde 2016, la Federación Salud Mental Castilla y León lleva a cabo el Curso de Asistente Personal. Pese a que la asistencia personal no solo se basa en el apoyo entre iguales, dicho curso consiste en una formación y capacitación laboral teórico-práctica siguiendo el modelo del apoyo entre iguales. La formación cuenta también con una evaluación final^109^.

Recientemente, entidades de Burgos han participado en el programa formativo ¡ACOMPAÑAME!, en el que también han participado entidades navarras y madrileñas, validado por un panel internacional de expertos e implementado desde el 2019. Como elementos novedosos, el curso incluye la realidad virtual, la robótica y una evaluación del programa por parte del dinamizador y los participantes. Además, cuenta con dos sesiones de evaluación: una inicial, que evalúa la situación cognitiva de las personas participantes y formaliza su participación en el curso y, una final, que incluye un nuevo test de las pruebas psicométricas de la evaluación y añade pruebas de evaluación del aprendizaje adquirido^109^.

En este territorio se han hecho contrataciones a través de la fundación Intras con categoría profesional de cuidador y cuidadora a través de centros especiales de empleo^112^.

#### Cataluña

Cataluña es el único territorio que cuenta con un plan de implementación concreto^113^, aunque aún no se ha puesto en funcionamiento. Existen experiencias en la implementación de una figura llamada agente de salud en ámbitos como el penitenciario o la atención a las adicciones, con competencias muy similares a las consideradas en este trabajo para los y las agentes de apoyo entre iguales^113^^,^^114^. Existe también cierta tradición de grupos de acompañamiento que están entre la ayuda mutua y el apoyo entre iguales^115^. En 2017, se realizó por primera vez una formación de formadores de apoyo entre iguales organizado y evaluado por la Universidad de Barcelona a través de un proyecto Marie Skłodowska Curie^22^.

Actualmente, hay dos programas formativos acreditados. Uno, organizado por la asociación EMILIA, heredera del ya mencionado proyecto europeo del mismo nombre^50^, en el Instituto Bonanova de formación profesional como unidades optativas dentro del programa oficial de Técnico en Atención a Personas en Situación de Dependencia. Esta formación tiene una edición anual y es reconocida al formar parte de un programa oficial. Por otro lado, la Universidad Central de Cataluña​​ en colaboración con las empresas proveedoras Althaia y Osonament llevan a cabo una formación organizada por profesionales para personas usuarias^116^ y ha iniciado colaboraciones con entidades “En primera persona” para darle continuidad.

En cuanto a la incorporación, algunas entidades proveedoras del sistema de salud han iniciado contrataciones, estableciendo convenios con entidades “En primera persona” sin ánimo de lucro, a través de figuras laborales como el técnico de acompañamiento, inicialmente pensada para el campo de la integración laboral de personas con discapacidad.

Cataluña está siendo, junto con Australia^117^, Inglaterra^25^, Dinamarca^83^y Quebec^118^, pionera en la implementación del apoyo mutuo en salud mental en poblaciones infantojuveniles. Actualmente, se está implementando un proyecto piloto dentro del Hospital de Día Infantil y Juvenil del Servicio de Psiquiatría del Hospital Clínic de Barcelona^25^. El proyecto plantea grupos de ayuda mutua con la colaboración de agentes de apoyo entre iguales, mayores de edad, que hayan experimentado sufrimiento psíquico en su juventud.

#### Euskadi

En Euskadi no se conoce la existencia de programas formativos específicos. Sin embargo, un miembro de la Asociación Guipuzcoana de Familiares y Personas con Problemas de Salud Mental (AGIFES) ha participado en el proyecto europeo *peer2peer*^54^ y la asociación ha incorporado pares con figuras laborales como las de monitor, comunes en los servicios de ocio.

#### Madrid

La Fundación Manantial, un proveedor de servicios de rehabilitación a gran escala, que incluye un extinto proyecto de Unidad de Atención Temprana basado en el diálogo abierto^119^, ha sido pionera en la incorporación de pares en la región. Se realizó una incorporación en el puesto de trabajo de experto por experiencia con la categoría profesional de educador. Esta incorporación no se mantuvo cuando la unidad fue absorbida por el Servicio Madrileño de Salud. En la actualidad, La Porvenir^120^, una entidad sin ánimo de lucro de reciente creación, cuenta con una par contratada. Por otro lado, entidades madrileñas han formado parte del programa formativo ¡ACOMPAÑAME! (ver Castilla y León)^109^ y recientemente se han organizado cursos de formación organizados por centros de salud mental de la red pública^121^^,^^122^.

#### Valencia

Desde 2019, la asociación sin ánimo de lucro ASIEM cuenta con un equipo técnico de apoyo mutuo entre iguales^123^. Este equipo desarrolla, por un lado, tareas de acompañamiento y seguimiento de personas usuarias de la red pública de salud mental y, por otro, tareas formativas para sus propias personas socias^124^.

#### Portugal

Portugal cuenta con un incipiente programa de implementación del apoyo entre iguales en salud mental para lo cual se ha desarrollado un documento de directrices prácticas^125^.

### El apoyo entre iguales como herramienta de desarrollo

Aparte del consorcio UPSIDES, que ya está implementando proyectos de formación e incorporación en India, Tanzania y Uganda^126^, otros proyectos internacionales como el QualityRights de la Organización Mundial de la Salud apuestan por el apoyo entre iguales como herramienta para fortalecer sistemas de atención a la salud mental dotados de pocos recursos^127^^,^^128^.

## DISCUSIÓN

El apoyo entre iguales en salud mental supone una herramienta que ha demostrado tener múltiples beneficios en el proceso de recuperación de las personas afectadas. Recientemente, también se ha avanzado en la demostración científica de su eficacia^46^. No obstante, en el entorno iberoamericano, la figura del agente de apoyo entre iguales o par aún está en una fase temprana de su proceso de implementación. 

El análisis llevado a cabo nos indica que los países germanoparlantes y Canadá son los que ofrecen programas de formación e incorporación más homogéneos. En el caso de países anglosajones y Francia, organizaciones sin ánimo de lucro se suelen hacer cargo de los programas formativos, a veces en alianza con instituciones académicas, aunque luego es posible la incorporación en el ámbito de los sistemas de salud, utilizando figuras laborales poco definidas. La situación en Latinoamérica, aunque incipiente, es esperanzadora, con experiencias formales en Argentina, Brasil y Chile y probablemente muchas otras informales. A nivel español, la implementación del agente de apoyo entre iguales también se encuentra en fases diferentes según la comunidad autónoma. Andalucía, Castilla-La Mancha, Cataluña y Valencia cuentan con programas formativos más o menos estables y el número de personas contratadas se encuentra en incremento. Por otro lado, encontramos Euskadi o Madrid, sin programas de formación definidos y con contrataciones puntuales.

El origen anglosajón del modelo de recuperación, base conceptual de la profesión de las y los agentes de apoyo entre iguales, además de la inclusión de estas prácticas en uno de los principales programas de salud mental global^128^, podría llevar a pensar que este tipo de prácticas son un tipo más de neocolonialismo. Claros argumentos a favor de esta idea son que los valores occidentales han impregnado tanto al modelo como a la profesión desde sus orígenes y las pulsiones neoliberales han condicionado su implementación^129^. Sin embargo, nos parece necesario destacar que, en un mundo donde las prácticas profesionales de la salud mental hace tiempo que se han globalizado, siguiendo un marcado patrón neocolonial y descontextualizador, cuyo máximo exponente es el Manual Diagnóstico y Estadístico de los Trastornos Mentales, las posibilidades de establecer diálogos entre saberes expertos y profanos debe ser aprovechada por los movimientos de personas afectadas y sus familiares. Estos deben potenciar una formación crítica de las personas que aspiren a ejercer el apoyo entre iguales profesionalizado para que puedan problematizar su nueva identidad profesional desde un prisma interseccional.

### Limitaciones

Una de las mayores limitaciones de este estudio es la posibilidad de que proyectos y experiencias con más capacidad para divulgar los resultados de sus evaluaciones, así como los que han participado en consorcios internacionales, estén sobrerrepresentados. Si bien es cierto que el objetivo de este trabajo ha sido recopilar información sobre el apoyo entre iguales profesionalizado, cuyas implicaciones institucionales suelen dejar huella digital, es posible que las iniciativas con carácter de base y cuya financiación tenga su origen en la economía social o se desarrollen de manera voluntaria puedan estar infrarrepresentadas.

### Recomendaciones en el contexto de su implementación en Cataluña

Teniendo en cuenta la evolución internacional y el panorama actual en el Estado español, podemos sacar algunas conclusiones y ofrecer algunas recomendaciones para la implementación de la figura en Cataluña. 

En cuanto a la legitimación de la formación recibida por las personas con experiencia como agentes, a corto plazo, se podría activar una unidad de competencia dentro de los procedimientos de evaluación y acreditación de las competencias profesionales adquiridas a través de la experiencia laboral o de vías no formales^130^. A largo plazo, consideramos que un escenario ideal sería la creación de un ciclo formativo oficial con reconocimiento sanitario. Por último, para la correcta integración de la figura en el sistema, en cualquier proceso de acreditación nuevo es muy importante establecer sinergias entre programas formativos y esquemas de incorporación que ya están en funcionamiento y asegurar la participación de sus promotores en las posibles comisiones de desarrollo futuro, además de la implicación de representantes de profesiones sanitarias reconocidas.

En lo que respecta a la incorporación de agentes de apoyo entre iguales, actualmente se puede llevar a cabo de forma directa desde entidades proveedoras sociosanitarias o entidades sin ánimo de lucro externas a los sistemas de salud, incluyendo entidades gestionadas por personas afectadas. Esto es posible a través de figuras contractuales que no exigen formación profesional o universitaria concreta. Sin embargo, la incorporación por parte de entidades proveedoras del Sistema Nacional de Salud tiene limitaciones derivadas de la Ley de Ordenación de Profesiones Sanitarias y la poca flexibilidad de los sistemas autonómicos en cuanto a las profesiones cuya contratación está permitida. A corto plazo, sin perder de vista que la situación ideal es la integración en el sistema sanitario, una posible opción sería que las entidades proveedoras de asistencia sanitaria subcontraten a entidades sin ánimo de lucro externas al sistema y que sean estas las que ofrezcan un contrato directo a las y los agentes. Por supuesto, otra posibilidad es incorporar personas que previamente se hayan formado como sanitarias a cualquier nivel, aunque esto plantea la necesidad de que estas personas revelen su experiencia. A modo ilustrativo, en países con mayor implementación de estas figuras como EEUU, es habitual que el personal facultativo o de enfermería con experiencia de sufrimiento psíquico ejerzan la coordinación de equipos de agentes de apoyo entre iguales. Esto facilita la legitimación por parte del resto de profesionales de estas instituciones, aunque plantea escenarios alejados de la horizontalidad.
