# Supplementary material for: Implementación de la figura de agente de apoyo entre iguales en salud mental: una perspectiva internacional en el contexto de su implementación en Cataluña
Source: Salud Colect. 2023 Jan 23;19:e4252. [Article in Spanish] doi: 10.18294/sc.2023.4252 (PMC11930321; doi:10.18294/sc.2023.4252)
Supplement: Translation

# Translation: Implementing the figure of peer support workers in mental health: an international perspective from the context of its implementation in Catalonia

**Authors:** Francisco José Eiroa-Orosa, Cecilia Sánchez-Moscona

**Affiliations:** 1 PhD in Clinical Pyschology and Psychiatry. Ramón y Cajal Researcher, Personality, Evaluation and Psychological Treatment Section, Department of Clinical Psychology and Psychobiology, Universidad de Barcelona. Member, Research Group Salud Mental en Primera Persona, Federación Veus, Barcelona, Spain. fjeiroa@gmail.com; 2 Master’s Degree in General Health Psychology. Personality, Evaluation and Psychological Treatment Section, Department of Clinical Psychology and Psychobiology, Universidad de Barcelona. Member, Research Group Salud Mental en Primera Persona, Federación Veus, Barcelona, Spain. csanmos@gmail.com

**Keywords:** Mental Health, Peer Support, Health Systems, Health Human Resource Training.

## Abstract

In the context of debates surrounding the training of mental health peer support workers and their incorporation into the Catalan Health System, this article presents a literature review complemented by interviews carried out between 2020 and 2021 with both international and Spanish experts. Based on the information obtained, content analysis of elements of their training and integration within the health system was performed. German-speaking countries offer the most homogeneous training and recruitment programs. In the case of English- and French-speaking countries, non-profit or third sector organizations are usually in charge of training programs and recruitment. Various experiences with training programs exist in the Ibero-American world, although they are not recognized as professional qualifications. Recommendations are offered for the development of this figure in Catalonia, which include advancing towards professional training with recognition as health care providers, as well as contracting options from both socio-health or health care providers or from third sector entities.

## INTRODUCTION

### The recovery paradigm

To understand how the phenomenon of incorporating peer support into mental health care systems emerges, we must place ourselves in the paradigm of recovery in the English-speaking world. *Recovery* as a movement and later as a model - we use the word *paradigm* to encompass both concepts - is the product of synergies among movements of people with lived experience of mental distress (also called people with experiences in the first person or people affected in Spanish-speaking countries, and, in relation to care systems, called service users, consumers or survivors), as well as relatives and professionals. It was defined in its initial conceptualization as a multidimensional, “deeply personal, unique process of changing one’s attitudes, values, feelings, goals, skills, and/or roles. It is a way of living a satisfying, hopeful, and contributing life even with limitations caused by illness.”^1^ With roots in the introduction of humanist measures after the French Revolution and the American Civil War,^2^ this movement proposed shifting the focus of mental health interventions beyond symptomatic remission. The use of the term recovery implied an intentional polysemy: it at the same time referred to a subjective and idiosyncratic process, to a new proposed objective of mental health interventions, and to a large-scale strategy of health services transformation.

The recovery paradigm was born, among other things, inspired by the evidence of symptomatic remission found in the pioneering Vermont study.^3^^,^^4^ The results of this longitudinal study, confirmed by evidence produced in the following decades, highlighted that just as total symptomatic remission is possible, even in people with very intense and lasting experiences of mental distress, a significant life can also be led despite being in the process of recovery, that is, while perhaps still experiencing “residual” symptoms.^5^

One proof of the early impact of the recovery movement is the appearance of the concept in the first US Surgeon General’s report on mental health in 1999.^6^ The report emphasized the participation of consumers and their relatives in the planning of services and the proposal of a model of care focused on the “restoration of self-esteem and identity and on attaining meaningful roles in society.”^7^

The movements of people with lived experience of mental distress, present since the 19th century England, have demanded, in different ways the humanization of care services. Except for the closure of the large hospitals and the deinstitutionalization of care, the most revolutionary proposals, embodied above all in the antipsychiatry movement, had only been marginally implemented. However, the emergence and institutional recognition of the recovery model made the role of service users in the care relationship a predominant issue of debate. In this context, the different alliances of organizations of professionals and people with lived experience of mental distress and their relatives were involved in the transformation and even appropriation of care systems. The proposal was to move from a vision that situated service users as passive subjects to be cared for by the system, to one in which they are considered subjects of rights and are encouraged to explore their strengths and support other people in this search.^8^

It should be noted that in Spain and Latin America there were some differences with respect to other Western European countries in the implementation of major mental health reforms. Due to the ultra-right dictatorships present in many countries during the 20th century, the deinstitutionalization of the psychiatric system did not begin until the 1980s or 1990s, with the development of resources alternative to large psychiatric hospitals.^9^ Subsequently, the arrival of influences from the recovery movement was limited to community rehabilitation services, and it was not until the late 2000s that it spread to other settings through specific projects.

In the case of Spain, the 2008 economic crisis and the related cuts in healthcare budgets led to the withdrawal of funds for many of these projects.^10^ Furthermore, the 2012 Draft Reform of the Criminal Code promoted by the state government, which proposed the introduction of special security measures for people diagnosed with mental disorders in the Criminal Code, provoked an immediate reaction from the main mental health organizations with representation of people with lived experience of mental distress. This turning point can be considered as a definitive catalyst for what is known as the “first-person” (*en primera persona*) movement. This movement has included, in addition to a complex constellation of associations, the federation En Primera Persona in Andalusia since 2009, and Veus (Voices), formed in Catalonia in 2014. Both autonomous communities were also pioneers in the implementation of campaigns against stigma at the regional level, becoming especially active territories in the fight for the rights of people diagnosed with psychiatric disorders, notwithstanding the growing activity in the rest of the country. Such activity has crystallized in alliances among the two federations and the rest of the “first person” associations, as well as a “first person” committee within the Mental Health Confederation of Spain, which itself was born of the relatives’ movement.

In Latin America there is a growing interest in the recovery model^11^ especially as a tool for institutional transformation^12^^,^^13^ and citizenship advocacy^14^ inspired by the process in the US. The promoters of the ideas of recovery in Latin America consider themselves part of the legacy of movements and ideas with local roots, such as the anti-asylum struggle of Nise da Silveira, the liberation pedagogy of the Brazilian Paulo Freire^15^ or the social psychology of Pichon-Rivière,^16^ pioneer along with Harry Stack Sullivan^2^ in the training of mental health service users as facilitators of the recovery of others.^17^

The implementation of these ideas in health management systems has not been exempt from controversy and the need for readjustments in the expectations of activists - if not downright disappointment. The British collective Recovery in the Bin is likely the organization to have best made explicit their criticisms. Its members accuse the recovery model of having been colonized and implemented with neoliberal bases (for example, hiding staff cuts behind “self-managed recovery plans”) in a decontextualized and homogenizing way that ignores the fact that many people will not be able to recover if social justice is not achieved.^18^

### Peer support

One of the distinctive elements of the recovery model - sometimes even confusingly used as a metonymy, as if the recovery model involved just the incorporation of peer support into the system^5^ - are interventions carried out by people with lived experience of mental distress.^19^ Examples are mutual aid (defined as the creation and moderation of self-help groups) and peer support (defined as the training and incorporation into the health system of people with lived experience of mental distress). Both mutual aid and peer support consist primarily of the support provided to people with lived experience of mental distress by people who have had similar experiences. It promotes recovery regardless of diagnoses or biomedical classifications and is based on theories that consider that social proximity fosters motivation,^20^ provides a reference for comparison^21^ and increases the understanding of one’s own situation.^22^ This type of accompaniment expands social networks and offers acceptance, support, understanding, empathy and a sense of community, which increases hope, autonomy, self-efficacy and the assumption of responsibilities.^23^ Specifically, because of its role within the system, peer support allows the adoption of valuable social roles, no longer restricted to the passive role of service user, but also that of role model.^24^ In addition, it can be implemented at low cost and maintains its usefulness combined with traditional professional practices.^19^ Although the implementation of mutual support has so far focused on adult populations, there are also starting to be programs focused on child and adolescent populations.^25^

A Delphi study identified the key elements of the training of this professional figure internationally called “peer”: lived experience as a resource, ethical practice, self-well-being, focus on recovery and communication skills.^26^ Once incorporated, the tasks performed by these people can be categorized as direct or indirect support.^27^^)^ The activities considered direct tasks are the defense of rights (providing information and support), connecting service users to resources (such as desired services), sharing common experiences, building community (connecting the person with programs that link them to the community), building relationships (based on trust), facilitating group activities, developing skills and objectives, socialization, and the development of self-esteem. Indirect tasks include administration, communication, supervision, training, receiving support, and obtaining and verifying information. In addition, the work of peer support workers also includes actions aimed at building relationships with professionals and legitimizing the role of the figure.^28^ In this way, the tools that peers require to perform their functions go beyond the experience of mental distress, including life experience of recovery and resilience, a respectful approach, genuine presence, modeling, collaboration, and engagement.^27^

### Difficulties and controversies regarding the implementation of peer support

Care systems have identified problematic issues associated with peer support. Examples are the difficulties of integration in health teams, the presence of negative attitudes towards the recovery model in other professionals, conflict and confusion of roles, the lack of policies and practices regarding confidentiality, organizational structures and cultures without sufficient definition, and the lack of support and networking.^29^^,^^30^ As strategies to improve these aspects, it is recommended that structures, policies and practices be established that guide the incorporation of peers and that help to define more clearly their role,^29^^,^^31^^,^^32^ as well as the supervision and accompaniment of other professionals, both those of peer support and typical clinicians.^33^^,^^34^

Within the movements of people with lived experience of mental distress, doubts about the implementation of peer support as a professionalized figure problematize the adoption of a role of “expert” or person who “possesses expert knowledge” in contraposition to one’s own lived experience or “profane knowledge.”^35^ In other words, if the inclusion of peers in the system was proposed as a spearhead to promote reforms through work that recognizes the value of experience and horizontality in treatment, but many of these peers end up adopting a professional identity similar in its verticality to that of traditional professionals, the only change that will have been made is in the person, who will have adapted to the system as it is.^32^ In this case, the inclusion of peers could even help uphold the existing care system, justifying that “changes” have been made when in reality everything remains the same. Additionally, it is also argued that maintaining an identity closely linked to the past as service users can distance peer support workers from a professional identity necessary for integration within professional teams.^36^ As the synthesis of a reflection regarding a pioneering experience in Argentina,^37^ the possible contribution of the incorporation of peers to mental health teams is posited as an aid to professionals in reflecting on the relational dimension of their work, and on their ways of treating and thinking about service users.

At the scientific level, the supposed scarcity of empirical evidence supporting the effectiveness and efficacy of peer support is one of the main arguments of people who do not support its implementation. However, there are a total of nine review and quantitative synthesis works,^38^^,^^39^^,^^40^^,^^41^^,^^42^^,^^43^^,^^44^^,^^45^^,^^46^ although it is true that six of them have been published since 2019. The approaches of these studies (for example, comparisons with other professionals, such as case managers, or between situations in which the figure is or is not added) and the variables they address, often classified around symptomatology, use of services or subjectivity (recovery, hope and empowerment) are very different. In all cases, without exception, reference is made to the low quality of the studies and high risk of bias. Recommendations range from fierce criticism of the implementation of professionalized peer support without clear evidence^40^^,^^45^, to neutrality^38^^,^^42^ or cautious support,^39^^,^^41^^,^^43^^,^^44^ with the notable exception of the most recent meta-analysis, of higher quality and including more studies, whose conclusions support the implementation of the figure with no more reservations than the commitment to higher quality studies (an ever-present tagline in practically any meta-analysis of psychosocial interventions).^46^ The most widespread conclusion in all these studies is that peer support would only have an effect on recovery, hope, and empowerment variables; but not symptomatology, use of services, or physical health^39^^,^^40^^,^^41^^,^^42^^,^^44^^,^^45^ with the exception of use of emergency services^38^ and depressive symptomatology.^43^ These conclusions are refuted by the most recent work, published in September 2022, which confirms efficacy in terms of symptomatology as well as recovery, hope and empowerment, although not functionality.^46^

The controversy surrounding the empirical evidence of peer support generates discussions even in regions such as North America, where implementation is advanced. In a 2018 opinion piece, the popular mental health law expert DJ Jaffe,^47^ now deceased, argued that many of the studies on the effectiveness of professionalized peer support have been conducted by the programs themselves, that they lack experimental rigor, and that they do not indicate the diagnosis or severity of the disorders of the people being treated. In this way, Jaffe argues that conclusions cannot be drawn regarding the value of this type of support in people with “severe mental disorders,” making a clear distinction with people with other severity levels. Likewise, referring to the comparison between peer support workers with professionals in similar roles that was carried out by the Cochrane collaboration,^38^ Jaffe raises the question whether the favorable results of some studies can be justified by the mere presence of a person regardless of his or her background or orientation. In addition, he criticizes that in most studies backing professionalized peer support, improvements are only reported in “soft measures” such as hope, self-esteem or empowerment, without mentioning the “hard, meaningful metrics” that according to the author would be the rates of homelessness, arrest, needless hospitalization, incarceration, suicide, and victimization.

In response to this article, a group of advocates for professionalized peer support led by Yale University professor Larry Davidson^48^ consider it inaccurate to talk about lack of evidence, since in 2018 more than 30 studies found positive results in a number of areas. By way of clarification, they state that there is a big difference between treating a disorder and helping someone who is in a recovery process, and that people living with significant mental distress require more assistance and support than can be provided at a strictly biomedical level. They consider that the fact that the deployment of peer support increases the use of crisis services and decreases hospitalizations, thereby increasing some costs,^49^ must be interpreted favorably. They believe that otherwise many of these people would have been disconnected from outpatient care and even if health costs decreased, social costs would increase significantly. Training and hiring people in recovery to support others benefits all parties in fragmented systems with scarce resources: service users receive support from professionals who foster hope and help them navigate the health system, these professionals have paid employment in a role that supports their own recovery, and the systems obtain qualified staff that drives the achievement of results associated with the recovery of a meaningful life and not with the reduction of symptoms. The role of the peer support worker does not include treating one’s own symptoms or replacing the functions of other professionals, but seeks to complement clinical care, without minimizing the importance of addressing aspects such as incarceration, hospitalization, or homelessness. Evidence in this regard emphasizes that peers help involve people in affective relationships; improve relations between service users and outpatient service providers, decreasing their use of emergency services and the cost of care; decrease substance use and increase hope, empowerment, self-efficacy, self-care, and quality of life.

Apart from these discussions taking place in North America, from the point of view of our incipient experience in Catalonia it seems important to us to emphasize that the requirement of demonstrating the effectiveness of a profession and not of its interventions is unprecedented in the field of health. Today no one would question the usefulness of a health profession; its concrete interventions are evaluated, not its existence in general. That is why we believe that with peer support the level of rigor should be similar: evaluate the interventions, not the existence itself. On the contrary, very different things are likely being conflated. Some approaches to peer work are likely to work better than others, but doubting the advantages of empowering people with lived experience of mental distress - regardless of the effect on variables considered “hard” - has important ideological implications in terms of justifying a coercive and vertical system.

### Objective

The aim of this work is to identify and describe the existing experiences of implementation of peer support in mental health at the international level, but with a special focus on Spain, since, apart from its academic value, we develop this work in the context of the preparation of possible implementation programs in Catalonia. A content analysis of documents and interviews on the implementation of this figure in the health systems of various countries is proposed. Finally, the article presents a series of short and long-term recommendations regarding the implementation of certified training programs and the process of incorporating peer support workers, as well as their integration into the mental health care system.

## METHODS

As a starting point, a literature review was carried out between 2020 and 2021 on the implementation of peer support in various countries using Google searches with the aim of finding gray literature, and the following databases with the aim of finding peer-reviewed articles: Google Scholar, Scopus, APA PsycINFO, and Medline. The search terms used included: mutual support, peer support, implementation, incorporation, integration, recruitment, and training. Searches were conducted in English, Catalan, French, German, Italian and Spanish. To be included in the review, the documents had to address the training and/or incorporation of the professionalized figure of peer support worker into one or more health systems. For reasons of completeness and space, we do not include information on mutual aid groups or other self-managed initiatives independent of health systems.

In parallel, with the aim of complementing the review in cases where information was missing, emails were sent to key actors in the implementation of peer support at the international level. Based on the answers obtained and using a snowball method, we proceeded to interview various experts (see acknowledgements) by phone or email about the type of incorporation and other factors related to the implementation of peer support in the health systems of their countries. Based on all the information extracted from these sources, a content analysis was carried out. The criterion for content extraction was that it provided information on training systems and incorporation into the health system of peer support.

All documents analyzed are referenced in the corresponding section. The list of people interviewed can be found in the acknowledgments section.

## RESULTS

The summary of the results can be found in Table 1. Several countries have peer support training programs with different levels of recognition and accreditation. However, the biggest challenge remains the incorporation of this figure within health systems

**Table 1:** Synthesis of content analysis results. Years 2020-2021.

Country or region	Training	Institutions accrediting training	Title	Incorporation
Canada	Certified course	NGOs	Peer support worker, recovery specialist	NGOs – Healthcare providers
USA	Certified course + State Exam	NGOs -Universities	Peer support worker, recovery specialist	NGOs – Healthcare providers
United Kingdom and Ireland	Course certified by the National Health System	NGOs - National Health System	Peer support worker, recovery coach	NGOs – National Health Service
Australia	Certified course	NGOs - Universities	Peer support worker, recovery specialist	NGOs - Healthcare providers
New Zealand	Certified course	NGOs - Universities	Peer support worker	NGOs - Healthcare providers
Italy	Heterogeneous. There are official courses.	NGOs	Esperti in supporto tra pari (expert in peer support)	NGOs - Healthcare providers
German-speaking countries	Official vocational training	ONG (ExIn), European consortium (UPSIDES)	Genesungsbegleiter (recovery companion), peer-begleiter (peer companion)	Healthcare providers
Scandinavia	Certified course	NGOs	Erfaringskonsulent (experienced consultant, Denmark, Norway), Kamratstödjare (support partner, Sweden)	NGOs - Healthcare providers
France, Belgium, and French-speaking Switzerland	Very heterogeneous, some certified courses	NGOs - Universities	Médiateur de santé pair (France), pair aidant (France, Belgium), expert du vécu (Belgium), pair praticien (Switzerland)	NGOs - Healthcare providers
Netherlands	Certified course	NGOs	Ervaringsdeskundigen (experts by experience)	NGOs - Healthcare providers
Argentina	Very heterogeneous, generally unofficial	NGOs	Agente de apoyo mutuo, par (peer support worker, peer)	NGOs
Brazil	Very heterogeneous, generally unofficial	NGOs	Trabalhador/a de apoio de pares (peer support worker)	NGOs - Healthcare providers
Chile	Very heterogeneous, generally unofficial	NGOs	Especialista en apoyo a pares (expert in peer support)	NGOs
Spain	Very heterogeneous, generally unofficial	NGOs	Agente, técnico/a de (acompañamiento y) apoyo entre iguales, apoyo mutuo (Worker or Technician of -accompaniment and- peer support or mutual support)	NGOs – National Health Service (very limited)

Source: Own elaboration.

There have been six international projects that deserve separate mention. Firstly, the project Empowerment of Mental Illness Service Users: Lifelong Learning, Integration and Action (EMILIA),^50^ part of the European Commission’s sixth research framework program, was implemented between 2005 and 2010. Teams from Bosnia-Herzegovina, Greece, Denmark, Finland, France, Lithuania, Norway, Slovenia, Spain, Sweden, Poland, and the United Kingdom participated. One of the activities of the project included the training of “expert” service users. Although this concept does not fully coincide with the definition we use in this work, it is one of the most recent precedents. The implementation of the project involved opening of two lines of workplace involvement: the “expert” person as a trainer of service users and as a mediator between health institutions and service users.^51^^,^^52^

Secondly, the projects Experienced Involvement (EX-IN)^53^ and Peer to Peer: A route to recovery of People with Mental Illness through Peer Support Training and Employment, shortened to Peer2Peer,^54^ of the Leonardo da Vinci program (currently integrated into the Erasmus+ macro-program) should be highlighted. The first, more focused on German-speaking countries, will be discussed later in the corresponding section for that geographic area. The Peer2Peer project, made up of teams from Austria, Spain, Scotland (United Kingdom) and Romania, was the first implementation at a European level of the North American peer support framework. As a continuing education project, its activities were very focused on the creation of a common training framework^55^ adapted from the Professional Development Award (PDA) in Mental Health Peer Support program developed by the Scottish Recovery Network, one of the organizations that led the consortium.

More recently, the Using Peer Support in Developing Empowering Mental Health Services (UPSIDES) initiative has established an international peer support research and practice community, through funding from the European Commission’s research framework program.^56^ The project is a five-year, six-country study which replicates and scales-up peer support interventions, generating evidence of sustainable best practice in high-, middle- and low-income countries (two sites each in Germany and UK; one site each in India, Israel, Tanzania, and Uganda).

Finally, the projects funded within the Erasmus+ program currently running are Peer Support+ which includes partners from Estonia, Iceland and the Netherlands, and TuTo3-PAT-Peer and Team Support, including partners from Germany, Belgium, Spain, France, Norway, Quebec (Canada), and Romania. The first is focused on the development of training materials in “experiential support” methodologies and was developed between 2020 and 2022. The second, inaugurated in 2022, aims to go beyond the results of the Peer2Peer project by defining a competency and training framework (for both peers and the teams into which they are integrated), facilitating professional recognition at the European and international level.

Next, we will present the current panorama of various countries in which the figure of peer support is in the process of implementation. We try to combine chronological and geographical order, discussing first, inasmuch as possible, regions where the appearance of the figure was earlier. In territories where implementation is recent, such as Iberoamerica, we follow an alphabetical order.

### English-speaking countries

#### Canada

In Canada, a pioneer in the implementation of peer support in the support system for people with disabilities, there is a project called Peer Support Canada.^57^ It is linked to the Canadian Mental Health Association which is responsible for professional peer certification, and the Canadian Mental Health Commission,^58^ which has published practice guides for peer support^59^ and monitors its implementation and impact on the health system.^60^

At the training level, Canada has an official program recognized by the main mental health providers at the federal level. This country has approved and established national standards of practice that include knowledge, competency, experience and code of conduct requirements to effectively provide peer support services.^57^ As for incorporation, there are several requirements that include having completed the training, undergoing background checks, and having carried out additional practice placements.

Furthermore, Canada has local initiatives such as the Ontario Peer Development Initiative (OPDI), previously known as the Consumer Survivor Development Initiative (CSDI), founded in 1991.^61^ This project provides solid training for peer support workers, as their experiences have shown the importance of establishing a minimum standard of such training, especially in rural areas.^62^

#### United States

The United States is another veteran in implementing peer support programs in its complex health care system. Most states have already established programs to train and certify peer support workers or are in the process of implementing a program, a University of Texas monitoring system shows.^63^^,^^64^ Training programs differ between states, but most are run by organizations managed by people with lived experience of mental distress and have their own certification exams. Thus, training programs are mandatory to obtain certification, and to be able to request reimbursements through Medicaid, the government insurance intended for people whose income and resources are insufficient to pay for medical care.^63^ The incorporation is carried out by specific agencies that recruit using generic paraprofessional labor categories that do not require licenses granted by the states, as is the case of the rest of health professions.

#### United Kingdom and Ireland

Peer support is a recognized profession in both Ireland and the United Kingdom, although differently in each. There are well-established *charitable bodies* that encourage training and incorporation such as the Mental Health Foundation or the Scottish Recovery Network. In Scotland, while at first peer support training was conducted by the US provider Recovery Innovations, an adaptation to its national context has taken place. To promote its durability and its adaptation to the Scottish reality, the Scottish Recovery Network worked with the Scottish Qualifications Authority in the development of a national accreditation.^23^ Thus, currently, training programs are carried out by independent organizations such as the Scottish Recovery Network, which is valid within the Scottish Credit and Qualifications Framework.^23^^,^^65^ This training consists of two units: one theoretical (recovery context), based on the concepts of recovery, empowerment, mutuality and the role of peer support workers; and another practical (developing practice), which aims to bring participants closer in a more applied way to all the knowledge, skills and values required for their practice.^66^ Incorporation is mostly carried out in the third sector, through non-governmental organizations (NGOs) and social care providers. In addition, the Scottish Recovery Network was involved in the aforementioned Peer2Peer project,^54^ which led to training programs elsewhere.

It should be noted that in the United Kingdom, peer support programs have also been developed for the child and adolescent population. An example is the Nottingham Healthcare NHS Foundation Trust, which has begun to develop a project of peer support among service users of its child and adolescent mental health services (CAMHS), obtaining satisfactory results.^25^

In Ireland, although the incorporation of peer support workers has been more recent, it is currently fully standardized, with certifications from the City of Dublin and the Atlantic Technology universities and recruitment possibilities in the health system.^67^ The mental health office of the National Health Service Executive has recently published an extensive report on its impact on mental health care services.^68^

#### Australia and New Zealand

In both countries the implementation of the profession is well advanced. Training programs are usually run by NGOs. Regarding recruitment, in both countries there are specific professional categories and there are job opportunities both through the third sector and mental health systems.

In New Zealand, peer support training is currently a level 4 certificate in the national qualifications framework. A new academic program at the Auckland University of Technology will commence in 2023, leading to a bachelor’s degree in Lived Experience Leadership. Additionally, a New Zealand national workforce development unit has published a document with a clear strategy for implementing peer support between 2020 and 2025.^69^

#### South Africa

In South Africa the implementation of the figure is still incipient. There are recent studies on the strengths and limitations of the process of introducing peers into the care system.^70^

#### Israel

Although not a formally English-speaking country, Israel is a country where peer support is deeply rooted, including an implementation coordinator in the Ministry of Health, following a training and incorporation model similar to that of English-speaking countries.^71^

#### Italy

Italy has its own model of community mental health with roots in the anti-psychiatry movements of the mid-20th century. Although it has similarities with the recovery model, Italy was a pioneer in the processes of deinstitutionalization and its model can be considered a precedent. In recent years in regions such as Lombardy, experiences of user training and peer support have been developed based on forms of collaborative management between cooperatives and the health system.^72^^)^ Currently, about one hundred workers provide their service in a dozen Lombardy mental health centers, paid directly with funds from the region or through agreements with cooperatives providing services.^73^ Other places of classic importance in the Italian community mental health model, such as Trieste, have also incorporated peer support workers.^74^ In 2021, the first National Congress of User and Family Experts in Peer Support [Conferenza Nazionale degli Utenti e Familiari Esperti nel supporto tra Pari] was held, which has led to the publication of a national document on peer support in mental health sponsored by the Ministry of Health that defines competencies and functions of the figure.^75^

#### German-speaking countries: Germany, Austria, and Switzerland

These three German-speaking countries have organizations derived from the aforementioned EX-IN project,^76^^,^^77^^,^^78^ implemented between 2005 and 2007. These countries have developed a program integrated into their vocational training systems. The curriculum of the program was developed cooperatively between mental health service users and professionals and researchers and trainers, and is led by certified trainers. Additionally, the figure has been officially integrated into the health service delivery systems of the three German-speaking countries. Adding to this solid line of training and incorporation is the more recent German arm of the UPSIDES consortium with presence in three German cities.^79^

#### Nordic countries: Scandinavia and Finland

Although implementation in these countries has materialized mainly in the 2010s, the five Nordic countries (Denmark, Finland, Iceland, Norway, and Sweden) have an extensive network of peer support workers and officially recognized training programs. In general, in all countries of the region, training and recruitment is done through independent organizations, but the peer support workers are integrated into social services and mental health care networks.

In Denmark there have been initiatives since the 90s, although until the 2010s there were no pilots of global implementation experiences in the most populated areas.^80^ In 2014, an extensive mapping of peer support service providers in all regions of the country was published,^81^ and in 2018 a proposal for a common training framework was made.^82^ In addition, an ambitious statewide support program has been implemented among young people experiencing anxiety and depression.^83^

Finland introduced in 2009 the mandatory participation of experienced experts and peer support workers in the planning and development of services as one of the main objectives of its National Mental Health and Substance Abuse Plan.^84^ In addition, the Open Dialogue model, which originated in the Finnish region of Lapland, but has been widely disseminated internationally in recent years, has included the inclusion of peer support workers in the teams.^85^ Iceland, the smallest both in size and population (just 370,000 inhabitants), has been fully dedicated to peer support since the early 2000s, with its own training program that is being strengthening with Dutch and Estonian partners in the context of the Erasmus+ Peer Support+ project.^86^

Since 2012, Norway has also had national guidelines supporting the introduction of peer support. With official training academies in several regions, a needs mapping,^87^^)^ a handbook for proper hiring,^88^ a network for the defense of interests and organization of activities,^89^ and a solid implementation through municipal funding, Norway is a country with a solid commitment to professionalized peer support with levels of impact on the care system comparable to veteran countries such as Canada.^90^

Finally, Sweden also has an official training and recruitment model^91^ promoted by the organization of service users the Swedish Partnership for Mental Health (NSHP for its acronym in Swedish) that has advocated for a project to integrate the figure in the health system since 2016.^92^

#### French-speaking European countries: France, Belgium, and Switzerland

The panorama in French-speaking countries is very heterogeneous.^93^^,^^94^ France has several training programs in different regions.^95^ There are several accredited training programs with recognition throughout the country, such as the *Médiateur de Santé/Pair* program carried out by the World Health Organization French Collaborating Center for Research and Training in Mental Health (CCOMS Lille), or the university degrees of the Université Paris 8 (Vincennes - Saint-Denis) and Université Paris 13 (Bobigny), Université Grenoble Alpes, Aix-Marseille Université or Université Claude Bernard Lyon 1.^96^ Other options include non-profit organizations such as the *Solidarités Usagers Psy* care network.

The training program of the WHO collaborating center is one of the most complete at the international level and had a pilot phase where a number of aspects were considered, from contracting and recruitment to the subjective experiences of peers and their teams.^95^ The training is carried out in two phases, both lasting one year. The first consists of a certified university program and the second involves the integration into teams through internships. The theoretical phase is carried out by the Université de Lille and training sessions are held in three regions. Practical training involves the 16 adult psychiatry services that host mediators/health partners in training. Enrollment for training is subject to incorporation into one of these 16 departments, so that participants in this program have a two-year professional contract, in the form of renewable fixed-term contracts. As part of that training, peers do full- or part-time internships and work alongside a team professional.

As for Switzerland, apart from the EX-IN model of the German-speaking region, the French-speaking region has an incipient implementation of the figure,^32^ with official training programs such as one lasting two years led by the non-profit organization Re-pairs.^97^ In the predominantly French-speaking Belgian regions of Wallonia and Brussels, multiple peer training and onboarding initiatives have been developed, involving a dense support network.^98^ There is a map of all organizations dedicated to peer support that includes 35 organizations at the time of this article’s publication, of which 12 are specialized in mental health.^99^ Three local organizations (the NGO En Route, the San Martín neuropsychiatric hospital and the Namur High School) lead the aforementioned TuTo3-PAT project of the Erasmus+ program, currently in execution.

#### Netherlands

In the Netherlands, the national Trimbos Institute and the national mental health organization GGZ Nederland designed a professional competency profile for peer support workers in 2013. In 2015, an official training curriculum was published.^100^ Dutch healthcare authorities are currently accrediting a recognized profession and formal position for staff working in healthcare settings.^101^

#### Romania

As discussed earlier, Romanian organizations participated and continue to participate in two of the major European projects regarding peer support, Peer2Peer (2013-2015) and TuTo3-PAT (2022-2025). Despite the success of the first, which involved training activities in Romania^102^ and the translation of the handbook into Romanian,^103^ there is no record of continuity of implementation plans.

#### Latin America: Argentina, Brazil, Chile

Argentina has had diverse experiences and reflections on the implementation of professionalized peer support since the early 2010s.^33^^)^ Examples are the NGO Proyecto Suma^33^^,^^37^ and the Fundación Bipolares de Argentina, which carries out a training program based on the materials developed by the Peer2Peer project.^104^ However, there does not seem to be a clear framework for incorporation into the health system.

Another country with peer training experiences is Brazil, in which different training initiatives exist.^105^ Although there is not yet an official system of legitimization of the peer figure, some independent organizations do so at their own discretion and may or may not be connected to government agencies or other organizations that may recognize and hire peer support workers. In some states, workers are hired by government organizations. However, although the process has been initiated, a specific category is not yet available within the Brazilian occupation classification system.

Chile, with experiences in research processes managed by people with lived experience of mental distress,^106^ is another Ibero-American country with experience in peer support, although apparently occasionally without coordination at the national level. There are experiences linked to non-profit organizations of associations of service users and their relatives^107^ in collaboration with professional groups.^108^

#### Spain

Within Spain, the main focus of this work, different experiences of training and incorporation of peer support workers can be found. Recently, a literature review was carried out regarding training experiences in peer support in mental health in Spain.^109^ Five peer support experiences were identified and will be explained in the corresponding geographical sections.

At the state level, the Mental Health Confederation of Spain has carried out two editions of the course “Informing in the first person,” which aims to provide tools and information to volunteer peers so that they can welcome and accompany other people with lived experience of mental distress in the organizations to which they belong. It also has an evaluation that is carried out at the end of the training and an assessment of the usefulness of the training six months after its completion.^109^

We will next look more closely at the autonomous communities in which the figure of peer support worker is in the process of implementation.

#### Andalusia

The first peer training implemented exclusively in Spain took place in Andalusia between 2010 and 2011. It was through the project “Peer support in the Mental Health Services of Andalusia,” which was part of the Second Comprehensive Mental Health Plan of Andalusia and was coordinated by the Andalusian School of Public Health and the Andalusian Mental Health Federation.^109^^)^ Also in Andalusia is the Federación En Primera Persona that supports the Peer Support Network [Red de Apoyo Mutuo] of Andalusia. The federation was involved in the Peer2Peer project^54^ led by the Castilian-Leonese Intras foundation and by the Scottish Recovery Network. The training program is planned in a practical and participatory way and has a mid-term evaluation and another at the end.^109^ Subsequently, short-term contracts have been made through federated associations in the Andalusian Health System.^110^

#### Castile-La Mancha

The Castile-La Mancha autonomous community was pioneer in integrating the professional figure of experts by experience in the labor market. Since 2018, the Expert by Experience project of the Fundación Sociosanitaria de Castilla-La Mancha has existed at the autonomic level.^111^ The objective of the project is to integrate people with lived experience of mental distress as professionals within the Psychosocial and Occupationa Rehabilitation Centers’ teams. A training program has been implemented that has led to hiring through the Extraordinary Plan for Employment in Castile-La Mancha. It should be noted that the training program has a final phase of supervision of the peer support workers hired by the Psychosocial and Occupational Rehabilitation Centers.^109^

#### Castile and Leon

This autonomous community joined the European project Peer2peer^54^ through the Intras Foundation. This organization has continued to carry out training activities both in Castile and Leon and in other communities such as Andalusia or the Balearic Islands. In addition, since 2016 the Castile and Leon Mental Health Federation has carried out a Personal Assistant course. Although personal assistance is not only based on peer support, this course consists of theoretical-practical job training and instruction that follows a peer support model. The training also has a final evaluation.^109^

Recently, organizations from Burgos have participated in the training program ¡ACOMPAÑAME!, in which organizations from Navarre and Madrid have also participated, validated by an international panel of experts and implemented since 2019. As novel elements, the course includes virtual reality, robotics, and an evaluation of the program by the facilitator and as well as the participants. In addition, it has two evaluation sessions: an initial one, which evaluates the cognitive situation of the participants and formalizes their participation in the course; and a final one, which repeats the initial psychometric tests in addition to evaluating the knowledge acquired.^109^

In this territory, contracts have been made through the Intras foundation using the professional category of caregiver through special employment centers.^112^

#### Catalonia

Catalonia is the only territory that has a concrete implementation plan,^113^ although it has not been put into operation yet. There is experience in the implementation of a figure called health worker in environments such as prisons or addictions care, with competencies very similar to those considered in this work for peer support workers.^113^^,^^114^^)^ There is also a certain tradition of accompaniment groups combining mutual aid and peer support.^115^ In 2017, for the first time, a training of peer support trainers was organized and evaluated by the Universitat de Barcelona through a Marie Skłodowska Curie project.^22^

There are currently two accredited training programs. One is organized by the Associació Emilia, derived from the aforementioned European project of the same name,^50^ in the institute for vocational training Bonanova as optional units within the official program of Technician in Care for People in Situation of Dependency. This training is carried out in year-long cycles and is recognized as part of an official program. Additionally, the Universitat Central de Catalunya and local health providers such as Althaia and Osonament carry out a training originally organized by professionals for service users^116^ and has initiated collaborations with first person organizations to give it continuity.

As for recruitment, some health provider organizations have initiated contracts establishing agreements with first person non-profit organizations, through figures such as accompaniment technician, initially designed for the labor integration of people with disabilities.

Catalonia has become, along with Australia,^117^ England,^25^ Denmark^83^ and Quebec,^118^ a pioneer in the implementation of mental health peer support in child and youth populations. Currently, a pilot project is being implemented within the Children’s and Youth Day Hospital of the Psychiatry Service of the Hospital Clínic de Barcelona.^25^ The project proposes mutual aid groups with the collaboration of adult peer support workers who experienced mental distress in their youth.

#### Basque Country

In the Basque Country, the existence of specific training programs is unknown. However, a member of the Asociación Guipuzcoana de Familiares y Personas con Problemas de Salud Mental [Guipuzcoan Association of Relatives and People with Mental Health Problems] (AGIFES) has participated in the European Peer2Peer project^54^ and the association has incorporated peers with categories such as instructor, common in leisure services.

#### Madrid

The Fundación Manantial, a provider of large-scale rehabilitation services including a now-defunct Early Care Unit project based on open dialogue,^119^ has been a pioneer in the incorporation of peers in the region. A position of expert by experience was incorporated using the professional category of educator. This incorporation was not maintained when the unit was absorbed by the Madrid Health Service. Currently, La Porvenir,^120^ a newly created non-profit organization, has a peer on staff. Additionally, Madrid organizations have been part of the training program ¡ACOMPAÑAME! (see Castile and Leon),^109^ and recently training courses have been organized by public mental health centers.^121^^,^^122^.

#### Valencia

Since 2019, the non-profit association ASIEM has had a peer support team.^123^ This team carries out, on the one hand, tasks of accompaniment and monitoring of service users of the public mental health network and, on the other, training tasks for its own members.^124^.

#### Portugal

Portugal has an incipient program for the implementation of peer support in mental health for which a document of practical guidelines has been developed.^125^

### Peer support as a development tool

Apart from the UPSIDES consortium, which is already implementing training and incorporation projects in India, Tanzania and Uganda,^126^ other international projects such as the World Health Organization’s QualityRights are committed to peer support as a tool to strengthen poorly resourced mental health care systems.^127^^,^^128^

## DISCUSSION

Peer support in mental health is a tool that has shown to have multiple benefits in the recovery process of people with lived experience of mental distress. Recently, progress has also been made in the scientific demonstration of its efficacy.^46^ However, in the Ibero-American environment, peer support work is still at an early stage of implementation. 

The analysis carried out indicates that the German-speaking countries and Canada offer the most homogeneous training and incorporation programs. In the case of anglophone countries and France, non-profit organizations are usually in charge of training programs, sometimes in alliance with academic institutions, although it is possible to later incorporate peers into health systems using loosely defined labor categories. The situation in Latin America, although incipient, is encouraging, with formal experiences in Argentina, Brazil, and Chile, and likely many other informal ones. In Spain, the implementation of peer support is also in different phases in each autonomous community. Andalusia, Castile-La Mancha, Catalonia and Valencia have more or less stable training programs and the number of people hired is increasing. However, in Basque Country or Madrid, there are no defined training programs and hirings are isolated.

The origin of the recovery model in anglophone countries, the conceptual basis of the profession of peer support workers, in addition to the inclusion of these practices in one of the main global mental health programs,^128^ could lead one to think that these types of practices are just another type of neocolonialism. Clear arguments in favor of this idea are that Western values have permeated both the model and the profession since its origins and neoliberal impulses have conditioned its implementation.^129^ However, we find it necessary to emphasize that, in a world in which professional mental health practices have long been globalized, following a marked pattern of neocolonialism and decontextualization, being the main exponent the Diagnostic and Statistical Manual of Mental Disorders, the possibilities of establishing dialogues between expert and lay knowledge must be taken advantage of by the movements of people with lived experience of mental distress and their relatives. These opportunities should promote a critical training of people who aspire to carry out professionalized peer support to ensure that they can problematize their new professional identity from an intersectional perspective.

### Limitations

One of the major limitations of this study is the possibility that projects and experiences with more capacity to disseminate the results of their evaluations, as well as those that have participated in international consortia, are overrepresented. While it is true that the objective of this work has been to collect information on professionalized peer support, whose institutional implications often leave a digital footprint, it is possible that grassroots initiatives whose funding originates in the social economy or are developed on a voluntary basis may be underrepresented.

### Recommendations in the context of its implementation in Catalonia

Considering the international evolution and the current panorama in Spain, we can draw some conclusions and offer some recommendations for the implementation of the figure in Catalonia.

Regarding the legitimization of the training received by people with experience as workers, in the short term, a competency unit could be created within the procedures for evaluating and accrediting professional skills acquired through work experience or non-formal channels.^130^ In the long term, we believe that an ideal scenario would be the creation of an official training cycle with health care recognition. Finally, for the proper integration of the peer support figure in the system, in any new accreditation process it is very important to establish synergies between training programs and already-established recruitment schemes, and to ensure the participation of promoters of this figure in possible commissions for future development, in addition to the involvement of representatives of recognized health professions.

Regarding the incorporation of peer support workers, it can currently be carried out directly with healthcare providers or non-profit organizations external to health systems, including organizations managed by people with lived experience of mental distress. This is possible through contractual categories that do not require specific professional or university training. However, the incorporation by providers of the National Health System has limitations derived from the Law on the Regulation of Health Professions and the little flexibility of the autonomic systems in terms of the professions whose hiring is permitted. In the short term, without losing sight of the fact that the ideal situation is the integration into the health system, a possible option would be for healthcare providers to subcontract non-profit organizations external to the system and for them to be the ones that offer a direct contract to the peer support workers. Of course, another possibility is incorporating people who have previously been trained as health workers, although this would require people to reveal their lived experience. By way of illustration, in countries with greater implementation of these figures, such as the United States, it is common for medical or nursing staff with lived experience of mental distress to coordinate teams of peer support workers. This facilitates the legitimization by the rest of the professionals of these institutions, although such a scenario is far removed from the horizontality sought by peer support.
